# Peer Review of “Development and Assessment of a Point-of-Care Application (Genomic Medicine Guidance) for Heritable Thoracic Aortic Disease”

**DOI:** 10.2196/64355

**Published:** 2024-10-08

**Authors:** Anonymous

**Keywords:** genomic medicine, point of care, thoracic aortic aneurysm, aortic dissection, decision support

*This is a peer-review report for “Development and Assessment of a Point-of-Care Application (Genomic Medicine Guidance) for Heritable Thoracic Aortic Disease”*

## Round 1 Review

### General Comments

This paper [1] highlights an important application of a point-of-care application in guiding clinicians in their management of patients. It will be of interest to the community served by the journal.

### Specific Comments

#### Major Comments

There needs to be some statistics on the “accuracy” of the application. For example, a number of expert clinicians in genomic medicine and its use (eg, 10) need to make clinical decisions without the use of the application, and then get 10 other clinicians who are not experts in genomic medicine to use the application and compare the level of agreement. A concordance study of this type needs to be done.

## Round 2 Review

### General Comments

With respect to my initial recommendation: There needs to be some statistics on the “accuracy” of the application. For example, a number of expert clinicians in genomic medicine and its use (eg, 10) need to make clinical decisions without the use of the application, and then get 10 other clinicians who are not experts in genomic medicine to use the application and compare the level of agreement. A concordance study of this type needs to be done.

### Specific Comments

#### Major Comments

This should not be beyond the scope of the manuscript. Without this information, the manuscript is not high impact but low impact or a niche interest. I recommend you pursuing this recommendation.
